# The 3 × 2 achievement goal questionnaire for recreational sport: Polish validation and athlete goal profiles in relation to psychological characteristics

**DOI:** 10.1186/s13102-026-01600-4

**Published:** 2026-02-13

**Authors:** Maciej Tomczak, Paweł Kleka, Łukasz Bojkowski

**Affiliations:** 1 Department of Psychology, Poznan University of Physical Education, ul. Królowej Jadwigi 27/39, Poznań, 61-871 Poland; 2 Faculty of Psychology and Cognitive Sciences, Adam Mickiewicz University, ul. Szamarzewskiego 89AB, Poznań, 60-568 Poland

**Keywords:** Recreational sport, The 3 × 2 achievement goal model, Validation, Personality traits, Achievement goal profiles

## Abstract

**Purpose:**

Studying motivational determinants in recreational sport is important because of their health benefits. The 3 × 2 achievement goal model, which was developed relatively recently, has been adapted for recreational sport; however, further validation is needed due to the limited number of existing studies. Therefore, the aim of this study is to validate the 3 × 2 Achievement Goal Questionnaire for Recreational Sport in the Polish cultural context and to identify selected personal determinants for achievement goals and their profiles among athletes.

**Materials and methods:**

A total of 345 recreational athletes (177 women and 168 men) with a mean age of 20.84 (SD = 1.94) participated in the study. They reported exercising recreationally on average of 3.42 times per week. The following measures were used: the 3 × 2 Achievement Goal Questionnaire for Recreational Sport, the Goal Orientation in Exercise Measure, the Generalised Self-Efficacy Scale, the IPIP-BFM-20 questionnaire, the Rosenberg Self-Esteem Scale, and the Hope for Success questionnaire.

**Results:**

The construct validity of the questionnaire was satisfactory (CFA model fit: CFI = 0.952, TLI = 0.939, RMSEA = 0.076. SRMR = 0.049), and the scale’s reliability was fully acceptable (α = 0.83–0.94). Women scored lower than men on the *self-approach* (d=-0.26), *other-approach* (d=-0.44), and *other-avoidance* scales (d=-0.22). It was also demonstrated that a group of athletes characterised by a favourable achievement goal profile, including elevated *task* and *self-approach* and low *other-approach* and *avoidance*, was characterised by higher intellect, agreeableness, self-efficacy and hope for success than the group with lowered *task* and *self* scores and elevated intra-group values on the *other-approach* and *avoidance* subscales.

**Conslusion:**

The present study supports the validity of the 3 × 2 achievement goal model in recreational sport within the Polish cultural context. High levels of intellect, agreeableness, self-efficacy, and hope for success promote beneficial achievement goals in recreational sport.

## Introduction

Physical activity is classified as one of the fundamental health-promoting behaviours. Its positive relationship with physical and mental health is highlighted. It affects, among others, cardiorespiratory fitness, the musculoskeletal, immune and endocrine systems, and metabolism [1–3]. Links are also found with self-efficacy, mood, body image, well-being, social ties and support, resilience, self-esteem and cognitive functioning [2, 4–6]. Its crucial importance is emphasised in disease prevention, the support of rehabilitation and therapeutic processes, and ultimately the enhancement of quality of life [7–9]. However, a significant portion of people remain physically inactive. Data indicate, for example, that young people often have levels of physical activity below those recommended for health, which is a cause for concern [10, 11]. Therefore, investigating people’s motivational dispositions towards physical activity, including participation in recreational sports, seems very necessary. Research into these factors is particularly important for the subsequent development of beneficial pro-health motivation.

Among motivational theories used to explain and predict functioning in sport and physical activity, Achievement Goal Theory (AGT) occupies a vital position [12]. Various approaches to motivational factors related to AGT have undergone different transformations, beginning with the still-utilised dichotomous goal orientation model assuming two orientations: task and ego [13–15], through the trichotomous approach [16] and the 2 × 2 model [17–19], to the 3 × 2 model [20]. The latter contains components (6 factors) concerning absolute, intrapersonal, and interpersonal standards for activity evaluation. The first of these groups includes the *task-approach*, which refers to striving for mastery and the performance of the task, and *task-avoidance*, which concerns avoiding failure in mastering and performing tasks. The intrapersonal aspect contains the *self-approach*, relating to better task performance than previously (in the past, typically, etc.), and *self-avoidance*, understood as avoiding worse task performance than previously. These task and self-goals derive from the mastery goals (task/learning goals). In turn, the interpersonal dimension contains the *other-approach*, concerning better task performance than others, and the *other-avoidance*, expressed as avoiding worse performance than others [12, 20]. This approach has relatively recently penetrated recreational sport [12, 21]. However, the number of studies in this area is still relatively small, not only with regard to validating the 3 × 2 model but also regarding the determinants of achievement goals. Given the health-promoting and quality-of-life-supporting nature of recreational sport participation, research in this area appears essential.

Therefore, this study aims to validate the 3 × 2 model among recreational athletes in the Polish cultural context and to identify selected psychological determinants of achievement goals. To our knowledge, two validated scales in Poland incorporate the 3 × 2 achievement goal model [22, 23], which have been used to study recreational and professional/high performance athletes. The same scales are often used to assess the motivation of high performance and recreational athletes. This makes it possible to compare these groups in terms of achievement goals measured using a single instrument. However, other scales constructed according to the 3 × 2 achievement goal model were not developed specifically for recreational sport. The 3 × 2 Achievement Goal Questionnaire for Recreational Sport by Lower and Turner were developed for use in this context [12]. This questionnaire achieved satisfactory validity and reliability indices. Initially obtained results using the 3 × 2 Achievement Goal Questionnaire for Recreational Sport indicate the usefulness of the 3 × 2 model in recreational sport, highlighting the relationship between *task-approach* goals and the perceived social, intellectual, and fitness benefits of participating in recreational sport [21]. These findings support the adaptation of the 3 × 2 model to recreational sport. An important issue regarding the applicability of the latest 3 × 2 model to sport is also the distinction between *task* and *self goals*. Individuals performing physical tasks may focus on completing and mastering the tasks and/or on their own progress [12, 24]. This differentiation can also allow a more precise description of competence-based behaviours of recreational athletes, which, in turn, enables a more accurate analysis of goals related to long-term engagement in physical activity. The validation of the 3 × 2 Achievement Goal Questionnaire for Recreational Sport in the Polish cultural context will significantly expand research and diagnostic opportunities by providing a new, useful tool.

It is assumed that personality traits constitute significant antecedents of achievement goal development. Personality traits are often considered to be more fundamental in relation to other constructs. Meta-analysis of school and work settings using the 2 × 2 achievement goal model has indicated that high conscientiousness, emotional stability (the inverse of neuroticism), extraversion, intellect (openness), and agreeableness are associated with more favourable achievement goals – mastery approach. Conversely, low conscientiousness, emotional stability, extraversion, agreeableness, and intellect are more frequently associated with unfavourable goal orientation – performance (other) avoidance [25]. However, the number of studies on recreational sports in this area is minimal, especially for the newer 3 × 2 model. To our knowledge, only Tomczak et al. [22] have studied these relationships using the 3 × 2 AGQ-S by Mascret et al. [24]. They found a slight positive correlation between emotional stability and *task-approach* and a slight negative correlation between agreeableness and the *other-approach*. Furthermore, given the need to study the determinants of achievement goals in recreational sport, we will also assess their relationships with other psychological characteristics important from the perspective of the 3 × 2 model’s validity and the studied questionnaire. Payne [26] argues that, beyond personality traits, significant antecedents of goal orientation include among others generalised self-efficacy and self-esteem. Self-esteem has been shown to relate positively to favorable learning/mastery goals and negatively to unfavorable performance avoidance [26]. In turn, a meta-analysis presented by Huang [27] in the academic context indicated that high levels of self-efficacy correlate moderately or even strongly with mastery goals. It can be assumed that in recreational sport as well, high self-efficacy may promote the development of favourable goals supporting the initiation and continuation of long-term physical activity for health purposes. It is important to further investigate factors such as hope for success and their relation with achievement goals. Hope reflects beliefs about strong willpower and the possibility of finding solutions [28, 29]. Tomczak et al. [30] demonstrated that hope positively relates to task orientation and mediates the relationship between personality traits and this goal orientation.

In addition, we will also identify groups of recreational athletes with different achievement goal profiles based on the 3 × 2 model, in relation to the psychological characteristics of athletes. Such analysis appears more valuable as it extends beyond typical, individual relationships between particular variables and concerns the athlete’s goal profile, considering the simultaneous configuration of different achievement goals. This enables more comprehensive motivation diagnostics in terms of structure, considering high, medium, and low achievement goal values collectively, both within groups aspects (relative to levels of other goals in each group) and between groups aspects (relative to the level of individuals from other groups). Profile analyses have been conducted in sport but have often considered the dichotomous model of achievement goals containing task (mastery goals) and ego e.g., [31–34]. Favourable goal profiles contain high levels of task goals, which are related to a focus on development, learning, and mastery and effective completion of tasks [14, 35]. In this study, profiles will be determined for the 3 × 2 achievement goals model and among recreational athletes. To our knowledge, there are no studies on 3 × 2 goal profiles in recreational sports, but it can be assumed that a beneficial profile will include high values for *self-approach*, *task-approach* and low values for *other-approach* and *avoidance*. *Task* and *self goals* derive from the mastery/task factor and are related to the motivation to complete tasks and improve one’s skills, which can significantly promote the initiation and continuation of physical activity. In turn, low values on the *other-approach* and *avoidance* scales are associated with a limited need to compare one’s results with others, which also seems to be important due to the low level of external motivation not related to the pure pleasure of practicing sports. Subsequently, relationships between the studied personality traits and other psychological characteristics and the obtained achievement goal profiles will be assessed.

## Materials and methods

### Participants

The study included 345 athletes engaging in recreational sport (who declared that they practice sport primarily for health and fitness improvement). The group consisted of 177 women and 168 men. The subjects were selected using convenience sampling from faculties related to sport, physical activity, and health from the Poznan University of Physical Education (a typical university of this kind in a large city of Poland). The participants’ age was M = 20.84, SD = 1.94. They reported exercising recreationally on average of 3.42 times per week. They primarily engaged in disciplines such as football, swimming, running, volleyball, basketball, dance, and strength sports.

### Questionnaires

Achievement goals were assessed using the 3 × 2 Achievement Goal Questionnaire for Recreational Sport – 3 × 2 AGQ for Rec Sport, developed by Lower and Turner [12]. Before use, the questionnaire underwent a translation procedure. A linguistic expert translated the questionnaire into Polish; another expert subsequently translated the questionnaire into English. Then, a group of experts (three sport psychologists and a translator) established the final Polish version of the questionnaire. At the beginning of the study (in the first phase of initiating validation studies), participants were asked to express any doubts regarding the questions. However, participants reported no concerns. The 3 × 2 AGQ for Rec Sport consists of 18 items concern of goals that you have for recreational sport, referring to 6 dimensions: *task-approach* (example item: *To master the aspects of my performance*), *task-avoidance* (*To avoid failing to master the task*), *self-approach* (*To perform better than I typically do*), self-avoidance (*To avoid performing worse than I typically do*), *other-approach* (*To perform better than others*), and *other-avoidance* (*To avoid performing worse than everyone else*). Three items represent each dimension. The participant’s task is to determine how much a given statement applies to them on a scale from 1 to 6 [12]. The questionnaire and norms are available from the authors of the study upon reasonable request.

To examine validity, the Goal Orientation in Exercise Measure (GOEM) questionnaire [36] was applied in the Polish adaptation by Tomczak et al. [9], which is designed to measure ego and task orientation in physical activity not related to high performance/professional sport. The questionnaire consists of 10 items (5 ego and 5 task), and the participant’s task is to rate (scale: 1–5) how much a particular statement applies to them.

Self-efficacy was assessed using the Generalised Self-Efficacy Scale (GSES) by Schwarzer and Jerusalem in the Polish adaptation by Schwarzer, Jerusalem, and Juczyński. The scale is unidimensional and contains 10 items. The participant’s task is to evaluate (scale: 1–4) how much the statement applies to them [37].

The Snyder scale [38] in the Polish adaptation by Łaguna et al. [29] was used to measure hope for success. This questionnaire determines the level of hope regarding problem-solving skills (pathways) and willpower (agency), which can be summed into a total score. The questionnaire contains 12 items, while calculations use eight: four for each component. The participant’s task is to evaluate (on a response scale from 1 to 8) to what extent a given statement refers to them. The total score was used [29].

Self-esteem was assessed using Rosenberg’s Self-Esteem Scale (SES) in the Polish adaptation by Dzwonkowska, Lachowicz-Tabaczek, and Łaguna [39]. It consists of 10 items and is unifactorial. The participant’s task is to evaluate (on a scale from 0 to 4) the degree of agreement with a given statement.

The IPIP-BFM-20 questionnaire was applied to assess personality, which examines five traits from the so-called Big Five approach: extraversion, emotional stability, conscientiousness, intellect, and agreeableness. The scale consists of 20 items. The participant’s task is to determine how much the statement applies to them on a scale from 1 to 5 [40].

### Procedure

Study participants were informed that their participation in the research was voluntary. Furthermore, it was emphasised that they could withdraw from the study at any time. They were also asked about health contraindications for participating in the study. Participants were additionally asked about their physical and mental state during the study and any other obstacles that might hinder or prevent them from participating. None of the participants reported any problems. The study was anonymous and had no time limit. It was conducted in accordance with the Declaration of Helsinki. The Bioethics Committee at Poznan University of Medical Sciences (Poland) verified the study description. The Committee stated that the study does not bear the characteristics of a medical experiment and, according to Polish law and GCP, is not subject to the Bioethics Committee’s opinion (Statement No. KB-273/22). The study instruction, in accordance with the Bioethics Committee’s recommendations, included a statement that participation in the study and submission of a completed questionnaire would be considered as the participant’s informed consent to participate in the study. Furthermore, the study is part of a broader research project on the measurement and psychosocial correlates of goal orientation in high-performance and recreational athletes in relation to their physical activity.

### Statistical analysis

To verify the construct validity of the Polish version of the 3 × 2 AGQ for Rec Sport, confirmatory factor analysis with the Satorra-Bentler correction [41] was applied. CFI, TLI, RMSEA, and SRMR were used to assess the model’s fit to the data. CFI and TLI above 0.90 were assumed to indicate satisfactory model fit values. Conversely, values of 0.95 and above indicate a perfect fit. RMSEA and SRMR below 0.08 are satisfactory [42, 43]. Similar to the authors of the original questionnaire version, the fit of other/alternative models to the data was also examined, such as the 2 × 2, trichotomous, and dichotomous models. Comparing these models with the theoretical 3 × 2 model will strengthen the construct validity examination of the scale. Models with the following factor arrangements were verified: a 2 × 2 model in which *task* and *self* items with the same valence were placed on a common factor, while *other subscales* were placed on their hypothetical factors; then a trichotomous model in which all items from *task* and *self subscales* were placed on one common factor, while items form *other subscales* were placed on their respective factors; and a dichotomous model in which all items from *task* and *self* subscales were placed on one common factor, while all items from *other subscales* were placed on a second common factor [12].

Scale reliability was also assessed using Cronbach’s alpha coefficient. To compare women and men in terms of achievement goals, Student’s t-test for independent samples was applied. Pearson’s r correlation coefficient was used to assess individual relationships between variables. K-means cluster analysis was applied using the Hartigan-Wong algorithm to identify achievement goal profiles among recreational athletes. The analysis was performed on standardized variables. In order to select the number of clusters, we used the silhouette and elbow methods and the substantive interpretability of the obtained clusters. The silhouette index indicated a global maximum of 2 clusters and a local maximum of 4 clusters, while the Elbow method indicated 4 clusters. We then analyzed the substantive criterion. Theoretical considerations enabling interpretation and the number of people in clusters were primarily considered. Divisions from 2 to 6 clusters were analysed. The 2-cluster division contains two groups differing in the level of all characteristics (low and high). The 3-cluster division contains three groups with high, average, and low levels of characteristics. Clusters here have more variable within-group profiles than in the previous division. However, these two divisions (into 2 and 3 clusters) have little interpretative and empirical value, as they resemble simple transformations of continuous variables into qualitative ones – two and three groups.

In the 4-cluster division, a cluster with relatively low levels of task and self subscales and higher levels of *other-approach* and avoidance subscales was obtain (higher level in within-group comparison and average in between-cluster comparison). Another cluster with elevated *task* and *self-approach* values, average *task* and *self-avoidance* values, and low *other-approach* and *avoidance* values was also identified. Additionally, there are two more clusters, one with high levels of all characteristics and another with low levels of characteristics (with within-group dominance of *task-approach* and *other-approach*). The smallest group consists of 37 people.

Subsequently, a 5-cluster division was performed. It is similar to the 4-cluster division. However instead of one cluster containing low *task* and self values and higher within group values of *other-approach* and *avoidance* subscales, two groups with relatively similar profiles were formed. In each of them, the values of *other-approach* and *avoidance* dominate within the group. They differ in terms of their location. One is closer to the mean values and the second to lower values of the variables studied. The smallest group in this division contains 18 people. Conversely, the 6-cluster division is quite similar to the 5-group division, with the main difference being the addition of a cluster with medium *task-approach* levels (high level in within-group comparison) and low levels of the remaining goals. The smallest group in this division contains 12 people.

Based on these analyses, the most interpretable division is into 4 clusters. It includes groups/types of athletes with clearly different profiles of motivation to achieve goals, in which it is possible to quite clearly distinguish combinations of profiles that are beneficial and less beneficial for functioning in recreational sports. In addition, the smallest group in this division has 37 people, which is significantly more than in the divisions into five and six clusters. Four clusters were also possible to select based on the Silhouette and Elbow methods. Analyses were conducted in R and jamovi.

## Results

### Construct validity of 3 × 2 AGQ for recreational sport

**Fig. 1:**
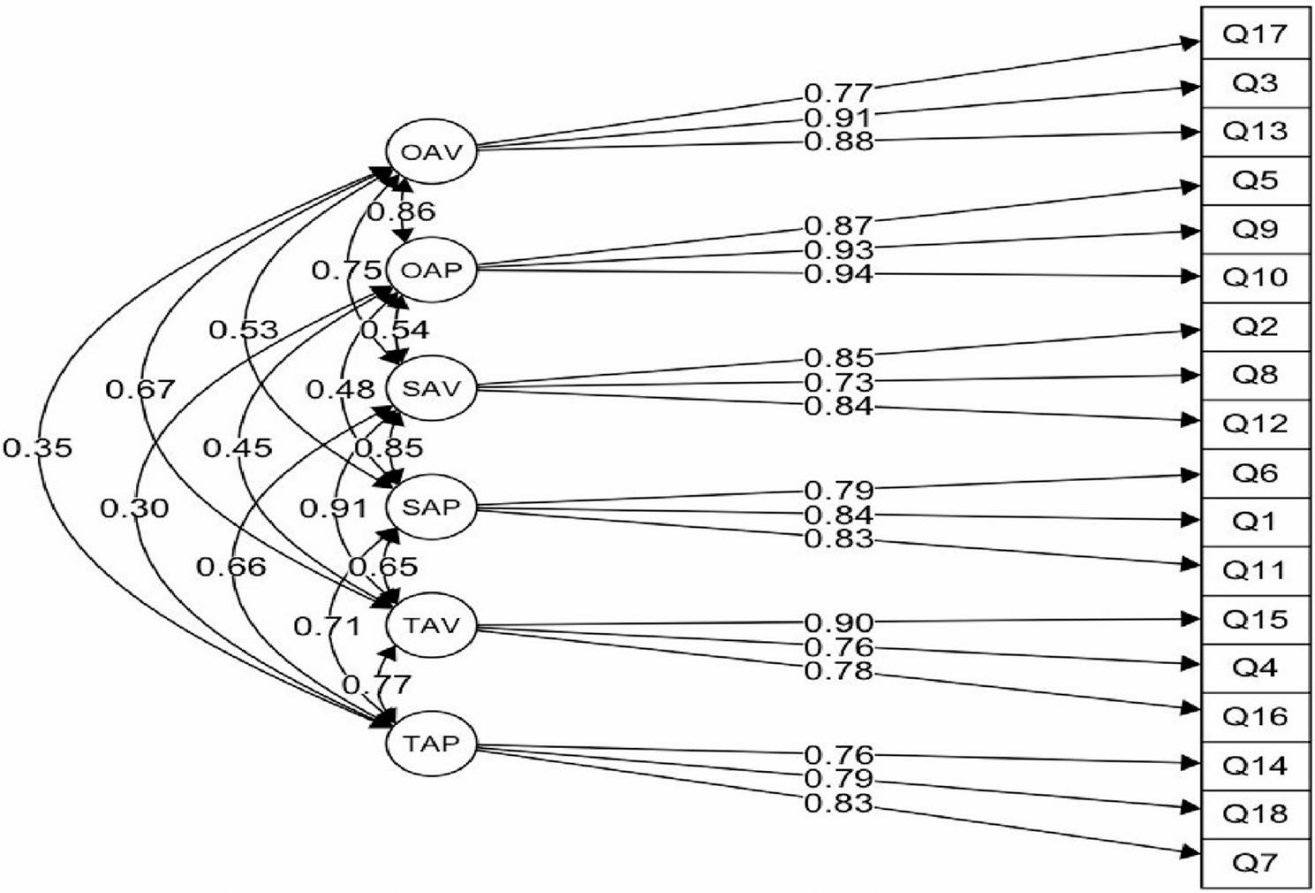
Factor structure of the 3x2 AGQ questionnaire for recreational sport – standardized solutions. Legend: TAP – Task approach, TAV – Task avoidance, SAP – Self approach, SAV – Self avoidance, OAP – Other approach, OAV – Other avoidance

The 3 × 2 model achieved a good fit to the empirical data. It was also superior to alternative models (Table 1; Fig. 1).

**Table 1:** Fit indices for the 3 × 2 model and alternative models

Fitting the 3 × 2 achievement goal model (1) and alternative models to the data							
Model	X^2^ / df		CFI	TLI	RMSEA	RMSEA 90% CI	SRMR
3 × 2 (1)	286.700 / 120		0.952	0.939	0.076	0.064; 0.087	0.049
2 × 2	462.450 / 129		0.904	0.886	0.104	0.093; 0.114	0.062
Trichotomus	590.132 / 132		0.862	0.840	0.123	0.113; 0.133	0.075
Dichotomus	661.962 / 134		0.829	0.805	0.136	0.125; 0.146	0.086

Legend: *CFI* Comparative Fit Index, *TLI* Tucker-Lewis Index, *RMSEA* Root Mean Square Error of Approximation, *SRMR* Standardized Root Mean Square Residual

### Reliability of 3 × 2 AGQ for recreational sport

Subscale reliabilities determined by Cronbach’s alpha and McDonald Omega coefficient are: *task-approach* – 0.83, 0.83; *task-avoidance* – 0.85, 0.86; *self-approach* – 0.86, 0.86; *self-avoidance* – 0.85, 0.85; *other-approach* – 0.94, 0.94; *other-avoidance* – 0.89, 0.89.

### Comparison of achievement goals in recreational sport between women and men

Significantly higher levels of *self-approach*, *other-approach*, and *other-avoidance* were observed among men. In other cases, no differences in achievement goals by gender were noted (Table 2).

**Table 2:** Comparison of achievement goal levels in recreational sport between women and men

Variables	Female M (SD)		Male M (SD)	t	*p*	Cohen’s d	Dif 95% CI
task.ap	15.06 (2.88)	15.24 (2.68)		-0.60	0.5456	-0.06	-0.182 (-0.753, 0.430)
task.av	13.54 (3.39)	13.80 (3.67)		-0.70	0.4834	-0.08	-0.266 (-1.030, 0.475)
self.ap	14.47 (3.54)	15.28 (2.79)		-2.36	0.0190	-0.26	-0.811 (-1.481, -0.146)
self.av	13.44 (3.53)	13.67 (3.53)		-0.595	0.5525	-0.06	-0.226 (-0.983, 0.521)
other.ap	10.55 (4.65)	12.54 (4.28)		-4.11	0.0000	-0.44	-1.982 (-2.900, -1.029)
other.av	11.34 (4.53)	12.34 (4.35)		-2.08	0.0384	-0.22	-0.995 (-1.947, -0.078)

Legend: *ap* Approach, *av* Avoidance, *Dif* Difference between means, *95% CI* 95% bootstrap percentile confidence interval

### Relationships between psychological variables and achievement goals among recreational athletes

Significant positive relationships were observed between the task subscale from the GOEM questionnaire and the *task* and *self* subscales from the 3 × 2 AGQ for Rec Sport. The strongest relationship occurred for the *task-approach* subscale. Significant positive relationships were also noted between the ego subscale and all subscales from the 3 × 2 AGQ for Rec Sport, with the strongest relationships were observed for the *other subscales*. Self-efficacy was positively related to *task-approach* and, to a lesser extent, to *task-avoidance*, *self-approach*, and *other-approach*. Hope for success was positively related to *task-approach* and, to a lesser extent, to *self-approach*. A marginally significant positive relationship between self-esteem and *task-approach* was also noted. Among personality traits, significant negative relationships were observed between agreeableness and *other-approach* and *avoidance*, as well as positive relationships between intellect and *task* and *self-approach*, and a positive relationship between emotional stability and *other-approach* (Table 3).

**Table 3:** Correlations between achievement goals and other psychological variables among recreational athletes

Variable	*N* = 345											
	task.ap		task.av		self.ap		self.av		other.ap		other.av	
	r	p	r	p	r	p	r	p	r	p	r	p
G.task	0.387	< 0.001	0.284	< 0.001	0.252	< 0.001	0.257	< 0.001	0.035	0.514	0.092	0.088
G.ego	0.167	0.002	0.277	< 0.001	0.231	< 0.001	0.308	< 0.001	0.550	< 0.001	0.495	< 0.001
SE	0.267	< 0.001	0.111	0.040	0.139	0.010	0.098	0.068	0.134	0.013	0.068	0.208
HS	0.259	< 0.001	0.065	0.230	0.124	0.021	0.051	0.348	0.089	0.098	0.010	0.855
SES	0.106	0.050	-0.047	0.383	-0.007	0.901	-0.037	0.496	0.058	0.285	-0.054	0.315
EXT	0.070	0.196	0.059	0.274	-0.032	0.549	0.029	0.593	0.052	0.337	0.034	0.531
AGR	0.067	0.217	0.004	0.935	-0.014	0.800	0.005	0.927	-0.153	0.004	-0.106	0.049
CON	0.072	0.185	0.085	0.115	-0.018	0.739	0.003	0.961	0.005	0.928	-0.008	0.880
EMS	0.061	0.258	-0.005	0.922	0.023	0.673	-0.019	0.721	0.159	0.003	0.042	0.432
INT	0.223	< 0.001	0.057	0.290	0.114	0.034	0.047	0.381	0.026	0.633	-0.035	0.521

Legend: *ap* Approach, *av* Avoidance, *G.task* subscale task from GOEM, *G.ego* subscale ego from GOEM, *SE* Self-efficacy, *HS* Hope for success, *SES* Self-esteem, *EXT* Extraversion, *AGR* agreeableness, *CON* Conscientiousness, *EMS* Emotional stability, *INT* Intellect

### Psychological characteristics and achievement goal profiles in recreational sport

**Fig. 2:**
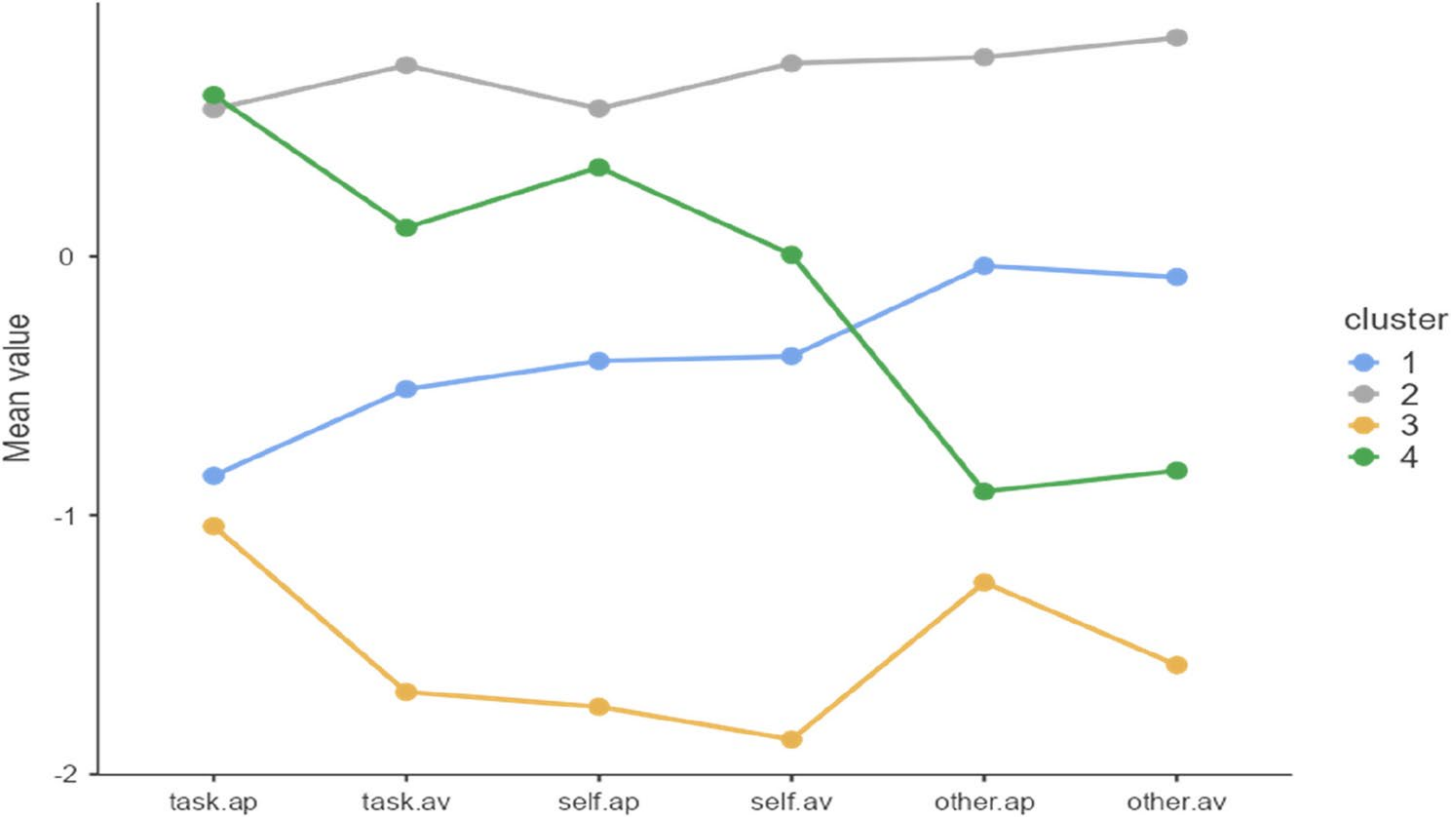
Achievement goal profiles among recreational athletes. Legend: ap – Approach, av – Avoidance

A division into 4 clusters was obtained. Cluster 1 included athletes with relatively low levels of *task* and *self goals* and higher levels of *other goals* in comparison with their *task* and *self* values, though this represents an average level when compared with the entire group. Cluster 2 included individuals with high values across all variables. Cluster 3 included individuals with low values in all goals, though in within-group comparisons, *task-approach* and *other-approach* goals dominate. Cluster 4, in turn, includes individuals with elevated *task-approach*, *self-approach* levels, average values of *task* and *self-avoidance* and relatively low scores on *other-approach* and *avoidance* goals (Fig. 2).

Significant differences in agreeableness, intellect, self-efficacy, and hope for success were observed among the identified groups (Table 4). Recreational athletes from cluster 4 are characterised by significantly higher agreeableness (Bonferroni: *p* < 0.05), intellect (*p* < 0.05), self-efficacy (*p* < 0.05), and hope for success (*p* < 0.05) than those from cluster 1. Additionally, athletes from cluster 2 are characterised by significantly higher levels of self-efficacy (*p* < 0.01) and hope for success (*p* < 0.05) than those from cluster 1.

**Table 4:** Comparison of psychological characteristic levels in the identified clusters

Variable	cluster 1	cluster 2	cluster 3	cluster 4	F, *p*, η^2^ ^df = 3.341^
	*N* = 99	*N* = 143	*N* = 37	*N* = 66	
	M (SD)	M (SD)	M (SD)	M (SD)	
EXT	3.40 (0.93)	3.47 (1.01)	3.38 (1.09)	3.36 (1.03)	F = 0.22, *p* = 0.883
AGR	3.75 (0.72)	3.81 (0.76)	3.93 (0.59)	4.06 (0.62)	F = 2.74, *p* = 0.043, η^2^ = 0.024
CON	3.27 (0.82)	3.32 (0.92)	3.14 (0.93)	3.43 (0.82)	F = 0.92, *p* = 0.432
EMS	2.73 (0.83)	2.90 (0.88)	2.75 (0.88)	2.60 (0.86)	F = 1.96, *p* = 0.119
INT	3.52 (0.75)	3.71 (0.66)	3.59 (0.73)	3.87 (0.75)	F = 3.46, *p* = 0.017, η^2^ = 0.029
SE	28.92 (4.33)	30.83 (4.41)	29.38 (4.60)	31.08 (3.76)	F = 5.33, *p* = 0.001, η^2^ = 0.045
HS	44.21 (7.17)	47.06 (7.32)	45.97 (8.24)	47.36 (6.78)	F = 3.69, *p* = 0.012, η^2^ = 0.031
SES	28.87 (4.44)	28.96 (5.92)	29.43 (5.40)	29.04 (5.47)	F = 0.10, *p* = 0.958

Legend: *SE* Self-efficacy, *HS* Hope for success, *SES* Self-esteem, *EXT* Extraversion, *AGR* Agreeableness, *CON* Conscientiousness, *EMS* Emotional stability, *INT* Intellect

## Discussion

First, the 3 × 2 Achievement Goal Questionnaire for Recreational Sport was validated. As part of the construct validity examination, confirmatory factor analysis was performed. The theoretically postulated model fit the empirical data well, significantly supporting the instrument’s validity. Furthermore, the 3 × 2 model proved to have a better estimated fit than alternative models such as the 2 × 2, trichotomous, and dichotomous models. Its fit according to CFI and RMSEA indices was better than that of the authors’ original scale version. Only SRMR indicated a worse fit than the original instrument version, although it was still good [12]. Better model fit to the data was also obtained than in the case of the 3 × 2 model estimated in the Polish version of the Mascret et al. [24] scale among competitive and recreational athletes [22] and a slightly weaker fit than that achieved by the Polish version of the scale developed by Wang et al. [44] in Rawat’s [23] validation. Furthermore, the fit of the model to the data from the Polish version of the 3 × 2 scale in recreational sports was relatively similar to the fit obtained by the authors of the original version of the scale tested among German students (Study 1), and worse than the fit of the model among US students (Study 2) [20]. It was also noted that men are characterised by higher levels of *other-approach*, *other-avoidance*, and *self-approach* compared to women. In the case of the other subscales, differences may result from men’s greater orientation toward competition and winning, which may relate to evolutionary and social aspects. This result is consistent with previous research findings e.g., [9, 36, 45]. Conversely, the result obtained for the *self-subscale* requires further research. On the other hand, fully satisfactory scale reliability indices were obtained.

Correlations with ego and task subscales from the GOEM instrument also support scale validity. Additionally, self-efficacy correlated with *task-approach* and, to a small extent, with *task-avoidance*, *self-approach*, and *other-approach*. Results for *task-approach*, *self-approach*, and *other-approach* regarding the relationship direction are consistent with Huang’s [27] meta-analysis results. However, the relationship strengths for *task-approach* and *self-approach* were smaller than those for the mastery factor from the meta-analysis. Conversely, the relationship with *task-avoidance* in particularl requires further analysis. Positive relationships between hope for success and *task* and *self-approach* were also demonstrated, consistent with predictions and results obtained by Tomczak et al. [22]. This is also consistent with Snyder’s [28] hope approach, according to which hope relates to positive goals. Furthermore, a marginal relationship between self-esteem and the *task-approach* subscale was demonstrated, consistent with Payne’s [26] meta-analysis results, where relationships between self-esteem and learning orientation were indicated. However, no relationship was observed between self-esteem and the *self-approach* scale, which, like *task-approach*, also originates from the mastery/learning approach factor. No relationships with the *other scales* were reported either, requiring further research. Relationships between personality traits and achievement goals among recreational athletes were also examined. Generally, it can be stated that not many relationships were noted compared to the results from the McCabe et al. [25] meta-analysis for school and work context. Few relationships between personality traits and achievement goals among recreational athletes were noted by Tomczak et al. [22]. The present study noted small positive relationships between intellect and *task* and *self-approach*, as well as small negative relationships between agreeableness and *other-approach* and *avoidance*. These results are broadly consistent with McCabe et al. [25] meta-analysis results. Additionally, a small positive relationship between emotional stability and *other-approach* was noted, which requires further research.

Subsequently, achievement goal profiles and their relationships with the studied psychological characteristics were analysed. Cluster analysis identified four groups with different goal profiles. Two clusters in particular draw attention – cluster 1 and cluster 4. In group 1, values for *other-approach* and *other-avoidance* that dominate compared to other factors in this cluster were noted (though compared to other groups, they had average values; they were higher than the values from two other groups). This group had relatively low *task* and *self-goal* values. Athletes from this group were characterised by significantly lower levels of agreeableness, intellect, lower self-efficacy, and hope for success compared to athletes from group 4, in which elevated *task-approach* and *self-approach* values dominated in the context of average *task-avoidance* and *self-avoidance* values and decidedly low *other-approach* and *other-avoidance* values. An elevated the *task-oriented approach* refers to a focus on the effective completion of tasks, while the self-oriented approach refers to a focus on one’s own progress in completing tasks. Low scores on the *other-approach* and *avoidance* scales indicate a low need to confront with others during task performance. It seems that this profile (group 4) is conducive to effective functioning in recreational sport, as it relates to desired motivation for engaging in health-promoting activities, favouring greater persistence in physical activity engagement. It is based on internal developmental needs instead of other/ego motivation related to comparing oneself with other recreational activity participants. Lochbaum et al. [46] indicate correlations of desired motivations, particularly with task and self-approach. It is worth noting that people from cluster 4 have certain trait values that may promote the development of a favourable goal profile. There are individuals with higher general self-efficacy and hope for success – possessing beliefs that they can cope with complex tasks, initiate goal-directed actions, seek solutions, and having the conviction about having strong willpower, may believe more in their capabilities and are generally more motivated to achieve goals [28, 29]. As the result, in the recreational sport environment, they may be more persistent in engaging in health-promoting physical activity while strengthening the development of favourable *task-approach* and *self-approach* goals. The observed higher agreeableness in cluster 4 may also relate to a lower likelihood of developing high levels of other/ego goals. Furthermore, this social dimension may favour the development of self and *task-approach.* It is possible that openness and tendencies to seek new solutions related to elevated intellect in this group (cluster 4) also favour the development of favourable achievement goals – *task* and *self-approach*. Conversely, the profile in which *other-approach* and *avoidance* subscales dominate compared to *task* and *self* goals within the group (cluster 1) is mainly related to competitive orientation as opposed to mastery, which relates to internal motivation for development and improvement. Individuals with profiles dominated by other/ego goals are mainly motivated by better results than others and avoiding worse performance than others [20, 24]. Such a profile does not contain an optimal set of goals enabling long-term engagement in recreational physical activity supported by internal motivation sources. Cluster 2 was also obtained, characterised by high levels of all goals. This group was characterised by significantly higher self-efficacy and hope for success than group 1, with dominant *other approach* and *avoidance* subscales within the cluster. The profile in cluster 2, alongside *other goals* (*approach*,* avoidance*), also contains high levels of desirable *task* and *self-approach* goals in sport. It is assumed that individuals from this cluster have, alongside other motivation sources, internal motivation for physical activity development. However, due to the high levels of other goals, it seems that this profile would be more adequate for competitive rather than recreational athletes.

The present study is not free from limitations. It would also be worthwhile to extend validation by examining test-retest reliability. Next, it is also worth verifying the invariance of the scale measurement in different groups of athletes, i.e., individual, team, older, younger, female, and male, and conducting validation studies in these groups. It is also worth assessing the achievement goal profiles in these groups. In addition, the results of our cluster analysis also need to be repeated in order to test the stability of the adopted solution. Another limitation of the study is the lack of consideration of more objective behavioural variables, including health-related variables. It is also worth examining the relationships between achievement goals and their profiles and more objectively measured physical activity. Assessing the relationships with these variables in subsequent studies could significantly strengthen the study’s validity and reinforce the investigation of achievement goal profiles as beneficial or less beneficial. In addition, the studies are cross-sectional in nature, which limits the drawing of causal conclusions. It is therefore worth verifying the results obtained through longitudinal studies, which will allow for better verification of the causal relationships between personality traits and the achievement goals of athletes. Another sample was selected by convenience sampling, and the study covered relatively young people who came from student groups. This limits the generalisation of the research results to the general population, older people, or less educated people. Furthermore, defining recreational athletes is often quite subjective and depends on the criteria adopted, which is also a limitation.

## Conclusions

In summary, the obtained results support the validity and reliability of the 3 × 2 AGQ for Rec Sport among Polish recreational athletes. Validation of the new instrument will significantly enrich research possibilities and enable diagnostics of recreational athletes in Poland. Furthermore, some relationships between personality traits and achievement goals from the 3 × 2 model were demonstrated. Conversely, the relationships that were noted generally had weak strength. Positive relationships between self-efficacy, hope for success, and self-esteem with selected achievement goals were also shown. Groups with different achievement goal profiles in recreational sport were also identified. It was demonstrated that the group of athletes with a favourable goal profile containing elevated *task-approach* and *self-approach* and low *other-approach* and *other-avoidance* scale values was characterised by higher levels of agreeableness, intellect, self-efficacy, and hope for success compared to the group with a less favourable goal profile, with low *task* and *self* values and elevated within-group *other approach* and *avoidance* subscale values.

## Data Availability

The datasets used and/or analysed during the current study are available from the corresponding author on reasonable request.

## Abbreviations

CFA: Confirmatory Factor Analysis
3x2 AGQ for Rec Sport: 3 × 2 Achievement Goal Questionnaire for Recreational Sport
IPIP-BFM-20: International Personality Item Pool, Big Five Markers, 20 items
GSES: Generalised Self–Efficacy Scale
AGT: Achievement Goal Theory
GOEM: Goal Orientation in Exercise Measure
SES: Self–Esteem Scale
CFI: Comparative Fit Index
TLI: Tucker–Lewis Index
RMSEA: Root Mean Square Error of Approximation
SRMR: Standardized Root Mean Square Residual
